# Awareness of product-related information, health messages and warnings on alcohol packaging among adolescents: a cross-sectional survey in the United Kingdom

**DOI:** 10.1093/pubmed/fdz080

**Published:** 2019-07-19

**Authors:** Nathan Critchlow, Daniel Jones, Crawford Moodie, Anne Marie MacKintosh, Niamh Fitzgerald, Lucie Hooper, Christopher Thomas, Jyotsna Vohra

**Affiliations:** 1 Institute for Social Marketing, and UK Centre for Tobacco and Alcohol Studies, Faculty of Health Sciences and Sport, University of Stirling, Stirling, UK; 2 Cancer Policy Research Centre (CPRC), Cancer Research UK, Angel Building, 407 St. John Street, London, UK

**Keywords:** alcohol labelling, alcohol packaging, health messages, health warnings, social marketing, young people

## Abstract

**Background:**

Alcohol packaging can be used to communicate product-related information, health messages and health warnings to consumers. We examined awareness and recall of such information and messaging among adolescents in the United Kingdom.

**Method:**

A cross-sectional survey was conducted with 11–19 year olds in the United Kingdom (*n* = 3399), with participants asked if they had seen any information, health messages or warnings on alcohol packaging in the past month (Yes/No) and, if so, what they recalled. We also assessed higher-risk drinking among current drinkers (≥5 Alcohol Use Disorders Identification Test-Consumption) and susceptibility to consume among never-drinkers.

**Results:**

One-third (32%) of participants had seen information, health messages or warnings on alcohol packaging. Chi-Square tests showed awareness was greater for current drinkers than non-drinkers (46% vs. 19%; *P* < 0.001), higher-risk drinkers than lower-risk drinkers (55% vs. 39%; *P* < 0.001), and susceptible never-drinkers than non-susceptible never-drinkers (21% vs. 16%; *P* = 0.01). Ten messages were recalled, with drinking responsibly (18%) and not drinking during pregnancy (13%) most recalled.

**Conclusion:**

Most young drinkers, including almost half of higher-risk drinkers, did not recall seeing any information, health messages or warnings on alcohol packaging in the past month, suggesting that current labelling is failing to reach this key audience.

## Introduction

In Europe, the proportion of young people aged 15–24 years old who are current drinkers, and the proportion that have engaged in heavy episodic drinking, is greater than in all other global regions.^1^ Although alcohol consumption among young people in the United Kingdom (UK) has been declining, approximately two-fifths of 11–15 year olds in England have consumed alcohol and almost a third of 16–24 year old drinkers have exceeded binge-drinking thresholds in the past week.^2^ The inclusion of health messaging and product-related information on alcohol packaging is a low-cost and high-reach intervention that may have the potential to moderate alcohol use and reduce higher-risk consumption among young people.^3,4^

In the UK, certain information is mandated on the packaging of alcohol products through national and international legislation on food and beverage standards.^5,6^ This includes the name and address of the supplier, country of origin, net quantity (in centilitres or millilitres), alcohol strength by volume (ABV, %), and use by date. The alcohol industry self-regulates other product-related information, health messaging, and warnings on packs. Examples include the number of alcohol units in the product, drinking guidelines (e.g. recommended units per week), warnings (e.g. liver disease and drinking during pregnancy), and messages concerning other alcohol-related harms (e.g. drink driving). Under this self-regulatory approach, such information is voluntary^6,7^ and there are no consistent standards of design or consistency on what information is required. For example, as part of the UK Government’s 2011 Responsibility Deal, the alcohol industry agreed to ensure that 80% of products displayed unit content, recommended lower-risk guidelines, and warnings on drinking during pregnancy.^8^ The latest guidelines, from 2017, no longer recommend the inclusion of lower-risk guidelines.^9^

Concerns have been raised over how health messaging and product-related information is communicated through alcohol packaging in the UK. For example, 2 years after the Chief Medical Officer issued revised drinking guidelines in the UK,^10^ an audit of over 300 alcohol products found that less than one-in-ten carried the revised guidelines, two-thirds referred to out-of-date guidance, and a quarter carried no guidance or guidelines.^11^ Even when drinking guidelines are present, research suggests that they are displayed in small fonts and positioned on the rear of the packaging (i.e. not in immediate eye line), and that other health messages are unclear or inconsistently formatted (e.g. warnings on drinking during pregnancy are smaller on wine than on beer products).^12^ Consistent with these concerns, research with adults in the UK has found that awareness of product information and health messaging on packaging is low, choices appear seldom informed by alcoholic unit content or drinking guidelines, consumers experience challenges in using labels to determine how many servings are equivalent to the recommended weekly limit, and current messaging and product labelling fails to capture attention.^5,13–15^

Research in the UK has only explored awareness of health messaging and product information on alcohol packaging among adult consumers. In this study, we explore awareness and recall of such information among adolescents and young adults aged 11–19 years old, what messages they recall seeing, and whether awareness differs by consumption group or demographics. Adolescents are important to investigate as alcohol use in this age group is linked to greater consumption and alcohol-related harms in later life.^16,17^ Therefore, exposure to product information and health messaging during formative experiences of alcohol may play an important role in shaping longer-term consumption.^18^

## Methods

### Design and sample

An online cross-sectional survey was conducted with 11–19 year olds in the UK in April-May 2017 (*n* = 3399). The survey was hosted by YouGov, a market research company, who recruited a sample designed to be representative of the UK population from their panel. A survey weight was provided for each respondent (based on age, gender, ethnicity, UK region, and social grade) to enable descriptive data to be representative of the UK population. Further details on the survey design and recruitment are reported elsewhere.^19,20^

### Measures

#### Demography

Demographic variables were obtained from information held by YouGov about panel participants or survey questions. Demographic variables included age, gender, ethnicity (recoded as White British and Other), resident country (England, Wales, Scotland and Northern Ireland) and quintile of deprivation (measured through Indices of Multiple Deprivation [IMD], a quantitative measure of local area deprivation based on elements such as income, crime and education).

#### Awareness of health messaging on alcohol packaging

Participants were asked ‘*Have you seen any information, health messages or warnings on alcohol packaging over the last month?*’ (Yes/No). No visual prompts or cues were provided, consistent with research measuring awareness of health messaging on cigarette packaging among young people.^21^ Participants who answered ‘Yes’ were asked ‘*What messages do you remember seeing?*’ with a free-text box for answers. Multiple answers were permitted in the free-text box, and participants were able to indicate ‘Don’t Know’ if they were unable to recall any messages.

#### Legal purchasing age and ever-drinking status

The sample was divided into those who met the minimum purchasing age for alcohol (≥18 years old) and those who did not. Participants were asked ‘*Have you ever had a whole alcoholic drink? Not just a sip*.’ Those answering ‘No’ were classed as never-drinkers while those answering ‘Yes’ were classed as ever-drinkers. A ‘Prefer not to say’ option was also provided.

#### Current drinking and higher-risk drinking status

Among ever-drinkers, alcohol consumption was measured through the Alcohol Use Disorders Identification Test–Consumption (AUDIT-C), a three-item scale which measured: (1) frequency of consumption (0 = Never to 4 = Four or more times a week); (2) number of units drunk in a typical drinking occasion (0 = One or two units to 4 = Ten or more units); and (3) frequency of heavy episodic drinking (0 = Never to 4 = Daily or almost daily). Heavy episodic drinking was defined as consuming six or more units if female, or eight or more units if male, in a typical drinking session; one unit of alcohol is equivalent to 10 ml or 8 g of pure alcohol. A cumulative AUDIT-C score was computed (0–12) and a cut-off score of ≥5 used to indicate higher-risk consumption.^22,23^ Participants who answered anything other than ‘Never’ to the first AUDIT-C item were categorized as ‘current drinkers’ and asked to complete items two and three (units drunk and frequency of heavy episodic drinking). Within current drinkers, the AUDIT-C had acceptable internal consistency (*α* = 0.79). All other participants were classed as ‘non-drinkers’ and were not asked to complete the final two items. The ‘non-drinker’ category includes never-drinkers and those who do not currently drink.

#### Susceptibility to consume alcohol

Participants were asked ‘*Do you think you will drink alcohol at any time during the next year?’* (1 = Definitely No to 4 = Definitely Yes; or Not sure). Never-drinkers were categorized as ‘susceptible’ if they answered other than ‘Definitely No’, while those who selected this option were categorized as ‘non-susceptible’.

### Ethics

Ethical approval was obtained from the University of Stirling General University Ethics Panel (GUEP59).

### Analysis

Data were analysed using SPSS version 23 and Microsoft Excel. All analyses were weighted to be representative of the demographic profile of the UK population. Frequencies examined awareness of product information, health messaging, and warnings on alcohol packaging in the past month. Pearson’s Chi-square tests examined differences in awareness by gender, ethnicity (White British vs. Other), resident country, legal purchase age status, ever-drinking status, current drinking status, higher-risk drinking status, and susceptibility. A Pearson’s Chi-square test also examined awareness by IMD, and a linear-by-linear association examined whether this increased from the more deprived to more affluent quintiles.

Responses to the free-text item were manually examined, and appropriate codes developed and refined from the raw data. An initial list of codes were developed by DJ, and revised and refined into 10 individual codes following discussion with NC and CM. Where participants provided information on more than one code (e.g. drinking during pregnancy and drink-driving), these were coded separately. Responses that were nonsensical or provided irrelevant information (e.g. messages about smoking or mention of receiving alcohol health education at school), examples of alcohol branding, and missing data, were excluded from analysis. If the branding also included product information, health messaging or warnings (e.g. ‘*Please enjoy Brand X responsibly’*) it was included. Following coding, weighted frequencies examined how often each code was recalled among those who had seen health messaging or product-related information on alcohol packaging within the past month, and how many different messages were recalled by each participant.

## Results

### Sample characteristics

The weighted sample (*n* = 3399) contained 51% males and an equal distribution across the five quintiles of deprivation (20% in each) (Table 1). Most participants were White British (76%), lived in England (84%) and were below the minimum legal purchasing age for alcohol (76%). The average age was 15.18 years old (*SD* = 2.55).

**Table 1:** Sample profile based on unweighted and weighted frequencies

	Unweighted		Weighted	
Variable	*%*	*n*	*%*	*n*
**Gender**				
Male	49	1679	51	1733
Female	51	1720	49	1666
**Ethnicity**				
White British	80	2716	76	2594
Other	19	647	23	779
Not specified or prefer not to say	1	36	1	26
**Country lived in**				
England	77	2601	84	2869
Scotland	12	424	8	265
Wales	7	250	5	160
Northern Ireland	4	124	3	105
**IMD Quintile** ^**a**^				
1 (most deprived)	20	680	20	676
2	20	666	20	676
3	21	723	20	676
4	18	616	20	676
5 (least deprived)	21	712	20	676
**Legal purchase age for alcohol**				
No	75	2551	76	2582
Yes	25	848	24	817
**Ever consumed alcohol** ^**b**^				
Never drinker	48	1598	49	1623
Ever drinker	52	1741	51	1713
**Current drinking status** ^b^				
Non-drinker^c^	52	1724	52	1747
Current drinker	48	1615	48	1590
**Higher-risk consumption** ^**d**^				
Lower-risk drinker	56	907	56	883
Higher-risk drinker	44	708	44	707
**Susceptible to consume** ^**e**^				
Non-susceptible	52	836	48	782
Susceptible	48	762	52	841

Cases excluded due to missing data:

^a^(*n* = 17 [weighted]).

^b^(*n* = 62 [weighted]).

^c^Non-drinker = Never consumed alcohol or do not currently consume alcohol.

^d^Base = All current drinkers.

^e^Base = All never drinkers.

### Alcohol consumption

After excluding cases with missing data on drinking status (*n* = 62, weighted), half of the weighted sample were ever-drinkers (51%). Almost half of the weighted sample were current drinkers (48%) and almost half of current drinkers (44%) were classified as consuming at higher-risk (≥5 on the AUDIT-C) (Table 1). Almost half of the weighted sample were never-drinkers (49%) and almost half of never-drinkers were classified as susceptible (52%).

### Awareness of messaging on packaging

Approximately a third of participants (32%) recalled seeing product-related information, health messaging or warnings on alcohol packaging in the past month (Table 2). Chi-square tests indicated that awareness was significantly greater in those above the legal purchasing age (48%) compared to those below (27%), *χ*^2^(1) = 128.53, *P* < 0.001, *ϕ*(Phi) = −0.19; in ever-drinkers (45%) versus never-drinkers (18%), *χ*^2^ (1) = 268.15, *P* < 0.001, *ϕ* = −0.28; in current drinkers (46%) versus non-drinkers (19%), *χ*^2^(1) = 294.40, *P* < 0.001, *ϕ* = −0.30; and in higher-risk drinkers (55%) versus lower-risk drinkers (39%), *χ*^2^(1) = 43.53, *P* < 0.001, *ϕ* = −0.17. In never-drinkers, awareness was greater among those susceptible to consuming alcohol (21%) compared to those not susceptible to consuming alcohol (16%), *χ*^2^(1) = 6.31, *P* = 0.01, *ϕ* = −0.06.

**Table 2:** Awareness of information, health messaging, and health warnings on packaging by demography and drinking status

	Aware of health messaging on packaging in past month		Chi-Square	
	*%*	*n*	χ^2^	*P*
**Weighted base**	32	1076	*-*	*-*
**Gender**			2.65	n.s.
Male	33	571		
Female	30	505		
**Ethnicity**			4.77	0.03
White British	31	798		
Other ethnicity	35	272		
**IMD Quintile**			16.95^a^	<0.001
1 (most deprived)	25	172		
2	30	203		
3	31	206		
4	37	250		
5 (least deprived)	34	227		
**Country lived in**			4.42	n.s.
England	32	921		
Scotland	31	81		
Wales	31	50		
Northern Ireland	23	24		
**Legal purchase age**			128.53	<0.001
Below legal purchase age	27	686		
Above legal purchase age	48	390		
**Ever consumed alcohol**			268.15	<0.001
Never drinker	18	295		
Ever drinker	45	764		
**Current drinking status**			294.40	<0.001
Not current drinker	19	324		
Current drinker	46	735		
**Higher-risk drinking** ^**b**^			43.53	<0.001
Lower-risk drinker	39	343		
Higher-risk drinker	55	392		
**Susceptibility** ^**c**^			6.31	0.01
Non-susceptible	16	123		
Susceptible	21	173		

Analyses are weighted

^a^Linear-by-Linear association.

^b^Based on current drinkers only.

^c^Based on never drinkers only.

Chi-square tests indicated that awareness was significantly lower in White British (31%) participants compared to Other ethnicities (35%), *χ*^2^(1) = 4.77, *P* = 0.03, *ϕ* = 0.04 (Table 2). There was also a significant difference between IMD quintiles (*P* < 0.001, *ϕ* = 0.08), with the linear-by-linear association indicating that those from more affluent quintiles reported greater awareness than those from lower quintiles, *χ*^2^(4) = 16.95, *P* < 0.001. Further analyses, however, suggested that these differences might partially be the result of the varied prevalence of alcohol use between the demographic groups. For example, those of White British ethnicity were significantly more likely to be current drinkers (51%) than those of Other ethnicities (36%), *χ*^2^ (1) = 51.85, *P* < 0.001, *ϕ* = 0.13. There was also a linear-by-linear association of current drinking across IMD quintiles, *χ*^2^(1) = 52.41, *p* < 0.001, *ϕ* = 0.16, with the most affluent quintile having a greater proportion of current drinkers (52%) than the most deprived (33%).

### Messages on packaging recalled

After removing nonsensical or irrelevant answers (*n* = 62, weighted) and missing data (*n* = 25, weighted), 41% of participants recalled one message, 9% two messages, 2% three messages, and 1% four messages (Table 3). The most commonly recalled messages related to drinking responsibly or in moderation (18%) and consumption during pregnancy (13%). For messages related to pregnancy, there was almost no difference in recall between males and females (12.4% and 12.9%, respectively). Messages recalled by the fewest participants included gender-related drinking guidelines (2%), daily drinking guidelines (2%), age limits for alcohol (1%), and product ABV (<1%). Just under half of respondents (47%) indicated ‘Don’t Know’ to what messages they had seen.

**Table 3:** Health messages on packaging recalled and weighted frequency of recall

	Frequency of recall	
	*%*	*n*
**Topic of health messages recalled**		
Drink responsibly	18	198
Don’t drink during pregnancy	13	135
Know and stick to your limits	7	71
Health, personal and social issues related to alcohol use	7	69
Don’t drink and drive	5	54
DrinkAware	5	56
Unit measurement	3	33
Gender drinking guidelines	2	24
Daily drinking guidelines	2	20
Over 18 only	1	14
ABV (%)	<1	4
**Total number of health messages recalled** ^**a**^		
None (answered don’t know)	47	460
One	41	410
Two	9	89
Three	2	23
Four	1	5

Data are weighted

Base: All participants who indicated they had seen health messaging on alcohol packaging in the past month.

^a^Excludes participants who provided a nonsensical or irrelevant answers (*n* = 62, weighted) and missing data (*n* = 25, weighted).

## Discussion

### Main findings of this study

Only a third of participants had seen any product-related information, health messages or warnings on alcohol packaging in the past month. Awareness was higher among those above the legal purchasing age, ever-drinkers, and current drinkers. This is to be expected given that non-drinkers and those who cannot legally purchase alcohol will typically have fewer opportunities to be exposed to alcohol packaging. Greater contact with alcohol packaging may also explain why higher-risk drinkers had the greater awareness. Nevertheless, less than half of those above the legal purchasing age (48%) or current drinkers (46%), and just over half of higher-risk drinkers (55%), reported being aware of information, health messages or warnings on alcohol packaging. Awareness was lower among those from less affluent quintiles and those not of White British ethnicity. The results, however, suggest that these differences may partially be a function of the varied proportions of drinkers within each demographic group.

Among participants who had seen health messaging or product-related information on alcohol packaging in the past month, the messages recalled most concerned drinking responsibly and drinking during pregnancy. The messages recalled least were for daily or gender-specific drinking guidelines, age-restriction messages, and product ABV. Of those who recalled seeing messages on packaging, most participants recalled only one message and almost half indicated that they did not know what messages they had seen.

### What is already known on this topic??

Research into other fast-moving consumer goods (e.g. tobacco and food) has shown that health messaging and warnings on packaging can promote healthier attitudes and behaviours.^24–30^ Research exploring similar information or messaging on alcohol packaging, however, has reported an inconsistent effect and gaps in the evidence.^31–35^ In the UK, there is limited information on alcohol packaging that is mandatory (e.g. ABV and product origin), with most messaging or warnings voluntary (e.g. drinking guidelines, unit content, drinking in pregnancy).^5–7^ Research which has examined current labelling practice suggests that alcohol packaging does not always carry up-to-date consumption guidelines, and that information is not highly visible and may be unclear or inconsistently formatted.^5,11,12,36^ Resultantly, adults report little knowledge of, and allocate limited attention to, the product-related information and messaging on alcohol packaging.^13–15^ There is no research exploring awareness among young people.

### What this study adds

This study shows, for the first time, the proportion of adolescents (above and below the legal purchasing age) in the UK that are aware of product-related information, health messaging, and warnings on alcohol packaging, and how this awareness varies by demography and consumption. That awareness was lower among young people from less affluent areas requires further exploration given the association this may have with health inequalities. That about half of current drinkers and those consuming alcohol at potentially higher risk were not aware of any health messaging or product-related information questions the nature and design of current labelling practices.

This is also the first study to consider what product-related information and messages young people recall from alcohol packaging. Ten different health messages were recalled, including factual product information (e.g. ABV and unit content), health messaging (e.g. consumption during pregnancy), and health or social issues related to alcohol (e.g. liver disease). Future research should explore the perceived relevance and efficacy of these messages among young people and the extent to which they inform their attitudes and consumption. Although several messages were recalled, only two were recalled by at least one-in-ten participants. The first was drink responsibly, a term considered strategically ambiguous as it is open to subjective interpretation, does not relate to an objective amount of alcohol or level of risk, and is often used to promote consumption of a brand (e.g. ‘*Please drink Brand X responsibly’*).^37–41^ The second concerned alcohol consumption during pregnancy, a message which may have limited efficacy to young people as the average age of first pregnancy in the UK is 28.8 years old.^42^ Seven messages were recalled by a minority of young people (one-in-twenty participants or fewer). This included messages around the age-restricted nature of alcohol and drinking guidelines.

Questions have been raised about the efficacy of self-regulation for alcohol marketing^43–46^ and packaging.^11,12^ In the UK, evidence suggests that current self-regulated labelling under-performs in comparison to more novel designs (e.g. pie charts showing proportion of weekly limit per serving)^15^ and does not always contain information that consumers consider informative.^5^ The suggested weak designs and poor clarity of self-regulated messages may help explain the low awareness and recall among young people. In the UK, health messages and warnings are mandatory on tobacco products,^47^ and research shows that such messages are influence smoking attitudes and behaviour.^25,26,48^ The current findings therefore suggest that further steps are required to increase the visibility and comprehension of messages on alcohol packaging, for example further exploration of optimal designs and standardizing across products.

### Limitations of this study

Although product-related information, health messages, and warnings may all shape consumption behaviour and attitudes, they are heterogeneous in design and the information provided. In this study, however, they were measured through a single combined item and no visual or written prompts were provided. Future research could therefore consider prompted and unprompted awareness of the individual components (e.g. drinking guidelines and pregnancy warnings). The awareness of information and messages reported in this study is also not indicative of salience or perceived credibility among young people. Future research is needed to explore young people’s understanding of and engagement with product-related information and health messages. The free-text item on messages recalled was not mandatory, and thus it cannot be determined whether ‘Don’t Know’ responses reflected a genuine uncertainty over which messages had been seen or a desire to minimize response time. Consequently, it is possible that the free-text responses are not exhaustive of all health messages seen by young people on alcohol packaging. Finally, while the initial question asked about product-related information, health messages and warnings, the subsequent free-text item only asked what ‘messages’ they recalled. Consequently, this may have led some respondents to not report relevant product information (e.g. ABV) or warnings (e.g. liver damage).

## Conclusion

This is the first study to examine awareness of product information, health messages and warnings among a demographically representative sample of adolescents in the UK. The findings show that most young people, including around half of current drinkers and half of higher-risk drinkers, did not recall seeing such information in the past month. Recall of messages was also low, with almost half of young people indicating they were unsure what messages they had seen. Further steps are needed to increase the visibility and comprehension of product information, health messages, and warnings on packaging.
